# Improving carbon monitoring and reporting in forests using spatially-explicit information

**DOI:** 10.1186/s13021-016-0065-6

**Published:** 2016-10-26

**Authors:** Céline Boisvenue, Byron P. Smiley, Joanne C. White, Werner A. Kurz, Michael A. Wulder

**Affiliations:** Pacific Forestry Centre, Canadian Forest Service, Natural Resources Canada, 506 West Burnside Road, Victoria, BC Canada

**Keywords:** Forest, Emissions reporting, Carbon, Greenhouse gas, Landsat, CBM-CFS3, Terrestrial ecosystem modelling

## Abstract

**Background:**

Understanding and quantifying carbon (C) exchanges between the biosphere and the atmosphere—specifically the process of C removal from the atmosphere, and how this process is changing—is the basis for developing appropriate adaptation and mitigation strategies for climate change. Monitoring forest systems and reporting on greenhouse gas (GHG) emissions and removals are now required components of international efforts aimed at mitigating rising atmospheric GHG. Spatially-explicit information about forests can improve the estimates of GHG emissions and removals. However, at present, remotely-sensed information on forest change is not commonly integrated into GHG reporting systems. New, detailed (30-m spatial resolution) forest change products derived from satellite time series informing on location, magnitude, and type of change, at an annual time step, have recently become available. Here we estimate the forest GHG balance using these new Landsat-based change data, a spatial forest inventory, and develop yield curves as inputs to the Carbon Budget Model of the Canadian Forest Sector (CBM-CFS3) to estimate GHG emissions and removals at a 30 m resolution for a 13 Mha pilot area in Saskatchewan, Canada.

**Results:**

Our results depict the forests as cumulative C sink (17.98 Tg C or 0.64 Tg C year^−1^) between 1984 and 2012 with an average C density of 206.5 (±0.6) Mg C ha^−1^. Comparisons between our estimates and estimates from Canada’s National Forest Carbon Monitoring, Accounting and Reporting System (NFCMARS) were possible only on a subset of our study area. In our simulations the area was a C sink, while the official reporting simulations, it was a C source. Forest area and overall C stock estimates also differ between the two simulated estimates.

**Conclusions:**

Both estimates have similar uncertainties, but the spatially-explicit results we present here better quantify the potential improvement brought on by spatially-explicit modelling. We discuss the source of the differences between these estimates. This study represents an important first step towards the integration of spatially-explicit information into Canada’s NFCMARS.

## Background

Current levels of atmospheric carbon dioxide (CO_2_) are unprecedented in the last 20 million years [1]. These levels of CO_2_ and other greenhouse gases (GHGs) have caused changes in climate that in turn have had widespread impacts on human and natural systems [2]. The latest Intergovernmental Panel on Climate Change (IPCC) report (2014) confirms that the earth has warmed about 1 °C in response to rising GHG concentrations and that further warming is highly likely. The rate of CO_2_ increase in the atmosphere can be reduced by taking advantage of the process by which atmospheric CO_2_ accumulates as carbon (C) in vegetation and soils in terrestrial ecosystems and in harvested wood products. Forest systems are the largest terrestrial C sink, removing about one quarter of annual anthropogenic CO_2_ emissions [3, 4]. Understanding C exchanges between the biosphere and the atmosphere, specifically the process of C removal from the atmosphere, and how this process is changing or might change, is the basis for developing appropriate adaptation and mitigation strategies. Presently, boreal forest systems are estimated to be C sinks [3, 5, 6]. However, projected changes in environmental conditions may result not only in a reduction in the proportion of anthropogenic CO_2_ forests remove from the atmosphere, but also in forests becoming a source of GHG to the atmosphere (example, [6–8]). Accurate representation of the emissions and removals from forest systems is a key component of monitoring and predicting changes in the global C cycle.

Historically, forest information and statistics have largely relied on plot-based field measurements [9]. However, with recent technological advancements, and increasingly refined reporting requirements, other sources of information provide new opportunities. Acquiring and using data from satellites or aircraft has notably increased the information base for describing forests in the last decades; for example, trends in phenology at northern latitudes have been remotely observed [10], remotely-sensed observations have provided support for renewable energy decisions [11], and aboveground biomass has been estimated remotely [12]. Observations from different types of sensors (i.e., optical and radar) are being explored to improve forest monitoring (example, [13]). Large international initiatives, such as Group on Earth Observations (GEO), are coordinating efforts to build a Global Earth Observation System of Systems, or GEOSS. GEO’s Global Forest Observations Initiative (GFOI), aims to foster the sustained availability of observations for national forest monitoring systems, exploiting the growing potential of remote observations to support decision making. GFOI works with national governments that report to international forest assessments [such as the global forest resources assessment (FRA) of the Food and Agriculture Organization, FAO] and the national GHG inventories reported to the UN Framework Convention on Climate Change (UNFCCC) using methods of the IPCC.

Robust national forest monitoring based on objective observations is widely accepted as a pre-requisite for countries to participate in international forest C agreements. University and government research activities, as well as initiatives such as GFOI, have fostered the development of methods and applications to produce remotely-sensed information products of relevance to monitoring and reporting programs. However, as of yet few of these products have been integrated into the reporting structures they aim to support, such as the national GHG inventories reported to the UNFCCC. To address these considerations, we fully integrate a 30 m spatial resolution change detection product derived from remotely-sensed data (Landsat, [14]), and a spatial forest inventory [15], into the Carbon Budget Model of the Canadian Forest Sector (CBM-CFS3—[16]) the core model of Canada’s National Forest Carbon Monitoring, Accounting and Reporting System (NFCMARS—[17]). CBM-CFS3 has a community of users around the world with applications in many forest systems outside Canada (examples, [18, 19]) making our integration advances relevant to other regions.

The second largest forest biome after tropical systems, the boreal forests spans the higher latitudes through Canada, Alaska, Siberia, China, and Scandinavia [3, 20]. Canada represents about one-third of the total boreal forest [21]. The sheer size of these forests, coupled with the fact that boreal forests are expected to experience the greatest warming of any forest biome as global temperatures rise (IPCC 2014), means that climate-related changes here have the capacity to significantly impact the global C cycle [22]. Recent studies have also shown that these systems are demonstrating altered environmental conditions via changes in forest growth [23–26] and mortality rates [27]. However, the C balance of boreal forests is also affected by changes in disturbance regimes [28–32] and at present, it is not clear how these many changes will affect the net C balance of boreal forests [6]. Unlike other forest systems where direct human-induced disturbance such as deforestation and forest degradation dominate the GHG balance (example, in Mexico, see [33]), the GHG emissions and removals from the atmosphere from boreal forest systems seem to be driven by natural disturbances [34]. Data from 1959 to 1997 show that an average of ~2 million ha burned annually in Canada with high inter annual variability [35]. With rising temperatures, the frequency and intensity of natural disturbances, specifically fires and especially in boreal forests, are also expected to increase [36, 37]. Information on the location and extent of forest disturbances are therefore essential inputs for accurate estimates of the boreal forest C balance [38]. For the Canadian portion of the managed boreal forest that is under reporting obligation [39], the dominant disturbances are fire and harvest [31]. As disturbance from fire and harvesting have vastly different C consequences, the ability to distinguish the cause or type of forest disturbance occurring on the landscape through space and time is especially important [19].

Assembling consistent (e.g., scale, coverage, vintage) and standardized (e.g., categories, estimating protocols) information products for a large and multi-jurisdictional nation, such as Canada, is difficult. Collaborative efforts to assemble the best available data have already resulted in an operational, science-based reporting system for the 2.3 million square kilometers of managed forests of Canada [34] that directly supports reporting and policy development (examples, [40, 41]), with completed reviews from UNFCCC expert review teams. For such a vast expanse of land (>230 Mkm^2^), field-based information can only be part of the inputs required for GHG estimation procedures. The current model, CBM-CFS3, allocates disturbance events from a variety of spatial and aspatial sources to deplete forest stand C from a forest inventory stand list in each of about 540–634 spatial units (depending on reporting year) defined by forest management units and jurisdiction (example, [41]).

Some remotely-sensed data and processing methods allow for forest-change detection that is consistent with the resolution of forest land management (~30 m) [42] in a cost effective way over large areas [43]. As noted, fire and harvest are the most common disturbances in the boreal forests of Canada, and have the greatest impact on the emission estimates [6, 31, 34]. As stand-replacing disturbances, fire and harvest are also the most reliably distinguished changes using remotely sensed data and analysis techniques [44]. From a spatial monitoring and modelling perspective, models of forest growth are mature and capture well biomass increment, but do not account for forest depletions, which can result in large changes in biomass and impact the ability of forests to exchange gasses with the atmosphere. Therefore, stand-replacing disturbances need to be well captured by monitoring systems. By incorporating change-detection products into regional GHG accounting and monitoring programs, it will also be possible to improve estimates of pre-disturbance forest conditions and it is anticipated that the reliability of present estimates will be improved, thereby enhancing our knowledge and understanding of the status of the large C stores contained in, and emissions from, these northern forests.

Historically modelling and computational constraints had limited the spatial resolution at which C accounting was performed in most jurisdictions, including Canada. Efforts to improve many facets of monitoring and reporting systems such as NFCMARS and CBM-CFS3 (CBM-CFS3—[16]) are starting to alleviate those constraints, by enabling the ingestion of spatially-explicit data for modelling C estimates for an extremely large number of records. Here we incorporate a newly available pan-Canadian forest change product derived from time series of Landsat data [45] with change attributed to disturbance type [14] into the CBM-CFS3, for a test area within the boreal forests of Saskatchewan, Canada under reporting obligations (~13 Mha). We aim to demonstrate the capacity for increased inclusion of detailed data (from spatial, temporal, and categorical perspectives) into a regional GHG modelling approach and hypothesize that incorporating the refined disturbance estimates will result in reduced uncertainties in GHG balance estimates for boreal forests.

## Methods

### Study area

**Fig. 1:**
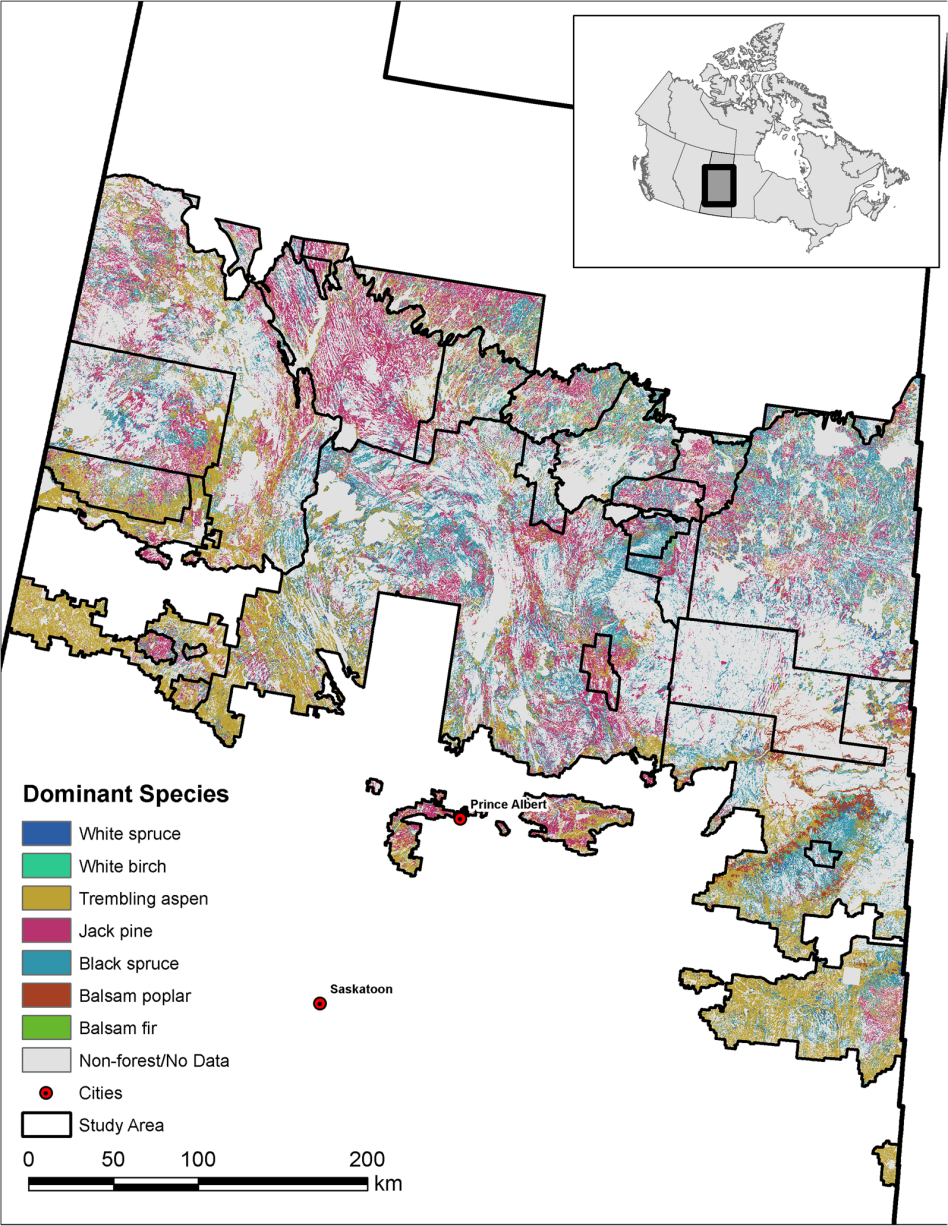
Our study area contains most of the forest subject to reporting obligations under the UNFCCC within the province of Saskatchewan, Canada. The southern regions of Saskatchewan are comprised of agricultural and grasslands, urban areas, and wetlands. *Coloured pixels* were the pixels modelled and show the species distribution as estimated from the spatial inventory dataset (CASFRI—15) for the beginning of the simulations (1984). The *black lines* show Forest Management Areas that comprise our study area

Seventy-five percent of all forests in Canada are boreal forests [21]. Our study area falls within the Boreal Plains and the Boreal Shield terrestrial ecozones of Canada (Ecological Stratification Working [46]). This forest is primarily composed of six main forest tree species: balsam fir (BF—*Abies balsamea*), balsam poplar (BP—*Populus balsamifera*), black spruce (BS—*Picea mariana*), jack pine (JP—*Pinus banksiana*), trembling aspen (TA—*Populus tremuloides*), white birch (WB—*Betula papyrifera*) and white spruce (WS—*Picea glauca*), with minor components of tamarack (*Larix laricina*), and Manitoba maple (*Acer negundo*). The climate in this region is characterized by predominantly short, cool summers and cold winters. The mean annual temperature ranges from −1 to 1 °C in the Boreal Plains and from −2.5 to −3 °C in the Boreal Shield with annual precipitation levels from 400 to 500 mm and 400 to 550 mm, respectively. Temperatures and potential evapotranspiration have increased in the region in recent decades, and soil moisture has declined [47, 48]. We combined a new spatially-explicit disturbance product, available spatially-explicit forest inventory, the best available productivity information, and improved modelling capabilities to the 5.9 Mha of forests that resided within the ~13 Mha in the managed forest zone in the province of  Saskatchewan (Fig. 1).

### Modelling

For our study, we have modified the input to CBM-CFS3, the core model to the Canadian reporting system (NFCMARS), to enable the use of spatially-explicit 30 m pixels instead of spatially-referenced forest stands, as the base unit for modelling. The CBM-CFS3 uses a combination of statistical modelling and process modelling to simulate forest C dynamics. Yield information compiled from forest inventory data and allometric equations are used to grow live biomass components on a yearly basis and process modelling is used to simulate dead organic matter pool dynamics. Biomass C includes all C in above- and below-ground living matter. Dead organic matter pools include hardwood and softwood snags and branches, all litter and organic horizons, as well as organic C in the mineral soil. More information on the model and its input requirements for use in NFCMARS are available in Stinson et al. [34] and the model is described in Kurz et al. [16].

### Model inputs

CBM-CFS3 simulations require information specific to each forest stand, such as age, dominant species, productivity level, and growth information. Simulations also require ecological parameters such as decomposition and turnover rates, general climate information as well as the timing, location, and type of disturbances.

#### Ecological parameters

Dead wood, litter, and soil organic matter C dynamics are explicitly simulated in CBM-CFS3, from the creation of snags to the decay of litter and dead wood and the eventual transfer of C into humified soil organic matter pools and the atmosphere. Annual turnover rates are specified for each of the above- and below-ground biomass pools tracked by the model, and when yield tables indicate declining biomass, the biomass C is transferred to the appropriate dead organic matter pools. Dead wood, litter, and soil organic matter decomposition rates are sensitive to mean annual temperature (using climate inputs developed after [49]), but no other climatic sensitivity is accounted for in this version of the model. We used the same turnover and decay rates that were developed for GHG balance reporting for the forests of Saskatchewan (see—[16, 34]).

#### Inventory

**Fig. 2:**
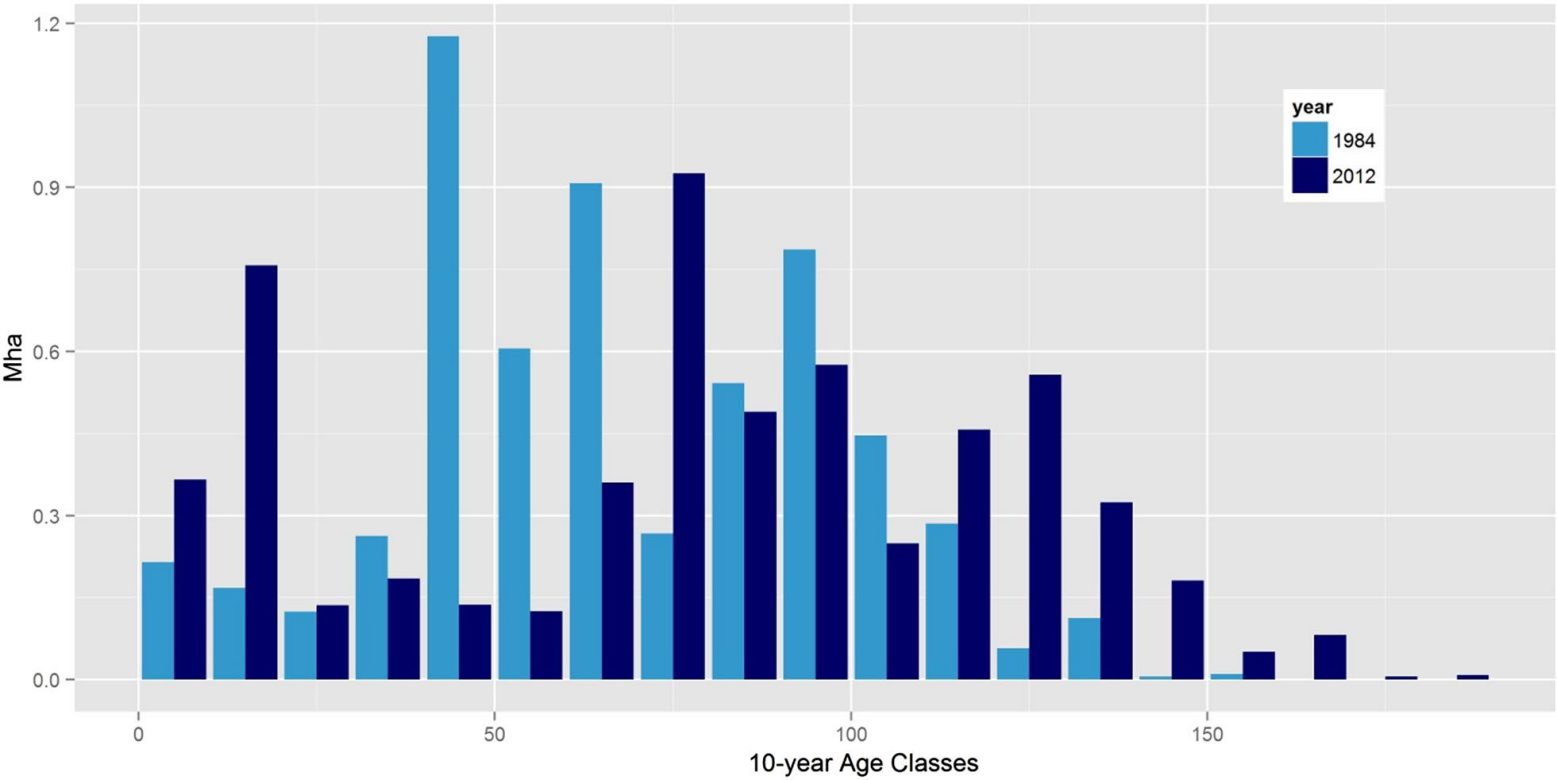
Age-class distribution in 10-year bins at the beginning of the simulations (1984) and at the end of the simulations (2012) according to CASFRI [15]

For our simulations, stand-level information was required for each pixel in our target area. Age, dominant species and productivity level were extracted from polygon information in Canada’s Forest Resource Inventories (CASFRI—[15]). CASFRI is a vector-based spatial forest inventory assembled for forest management and conservation projects (http://www.beaconsproject.ca/documents). CASFRI is a compilation of strategic forest inventories that are used by industry and government for resource management. In Canada, forest inventories are typically derived from interpretation of 1:10,000 or 1:20,000 scale stereo ortho-photography, where forest cover polygons are delineated and stand attributes are estimated by experienced air photo interpreters [50] and field-verified by forest inventory crews, following statistically-based sampling protocols. We used the photo-interpretation year to adjust the age and height of each pixel in our time series and resampled the vector-based data to a 30 m resolution to match our other spatial layers. We recompiled these data to produce information on initial forest conditions in CBM-CFS3 in 1984, the start of the simulation period, similarly to the procedure described in Sharma et al. [51]. Figure 1 shows the distribution of species at the beginning of the simulations, while Fig. 2 shows the age-class distribution in 1984 and at the end of the simulation in 2012. Inventory information was not available for all pixels in the managed portion of the forests of Saskatchewan (included in grey shades in Fig. 1).

#### Growth curves

Like many jurisdictions, Saskatchewan has a network of re-measured permanent sample field plots whose primary purpose is to inform growth and yield models for sustainable forest management [9]. Some of Saskatchewan’s permanent sample plots were established as early as 1949. The complete database contains 5313 measurements of 2048 plots. Using these data, we fit a linearized form of Hoerl’s function (Eq. 1a), a TYPE II combined exponential and power function [50] to develop the growth information needed for CBM-CFS3 simulations.

1a $${\text{Merchantable}}\,{\text{volume}} = \beta_{0} ({\text{age}})^{{\beta_{1} }} e^{{\beta_{2} ({\text{age}})}}$$

1b $$\log \left( {{\text{Merchantable}}\, {\text{volume}}} \right) = \beta_{1} \log \left( {\beta_{0} \left( {\text{age}} \right)} \right) + \beta_{2} \left( {\text{age}} \right) + \left( {1|plot} \right) + \varepsilon$$

where β_0_, β_1_, and β_2_ are parameters representing the intercept, the shape and scale of the growth model, (1|*plot*) represents the error structure (random effect) on the intercept due to plot remeasurements and the merchantable volume is in m^3^ ha^−1^.

Linearizing Hoerl’s function (Eq. 1b) simplifies the processes of finding the best parameters for our dataset. Permanent sample plots are re-measured through time and therefore violate the basic assumption of independence necessary for basic statistical fitting methods. Hence, to account for these data dependencies, we determined the long-term means of change in volume and its components with plot identity as a random factor to account for temporally autocorrelated plot-specific site condition and species composition. We estimated parameters with random effects (one intercept by plot—1|*plot*) and fixed effects using maximum likelihood by maximizing the joint density of the parameters and the random component. Adding the random effects in our model fitting lends structure to the random variation in volume measurements, accounting for plot re-measurements. Effectively, we split the error term in two. We assumed a normal distribution for both error terms with a covariance matrix equal to 0. The data were also stratified into nine strata defined by leading species and categorized by soil moisture regime from our spatial inventory information. The R-package lme4 [52] was used to fit this mixed-effect model.

#### Disturbances

Using the plentiful Landsat Thematic Mapper and Enhanced Thematic Mapper image archive for Canada [53], a best-available pixel image compositing approach was developed [54] that was in-turn used as an input to a time series-based change-detection algorithm [45]. A conceptual framework was developed followed by application of a disturbance attribution algorithm over the forested portion of the province of Saskatchewan [14]. Land-cover changes over the test area are detected with high overall accuracy (92.2%), with the majority of changes labeled to the correct occurrence year (91.1%) or within ±1 year (98.7%). Fire and harvesting events, the most important disturbances for C balance in these forest types, are the most successfully attributed (commission error <10%).

**Fig. 3:**
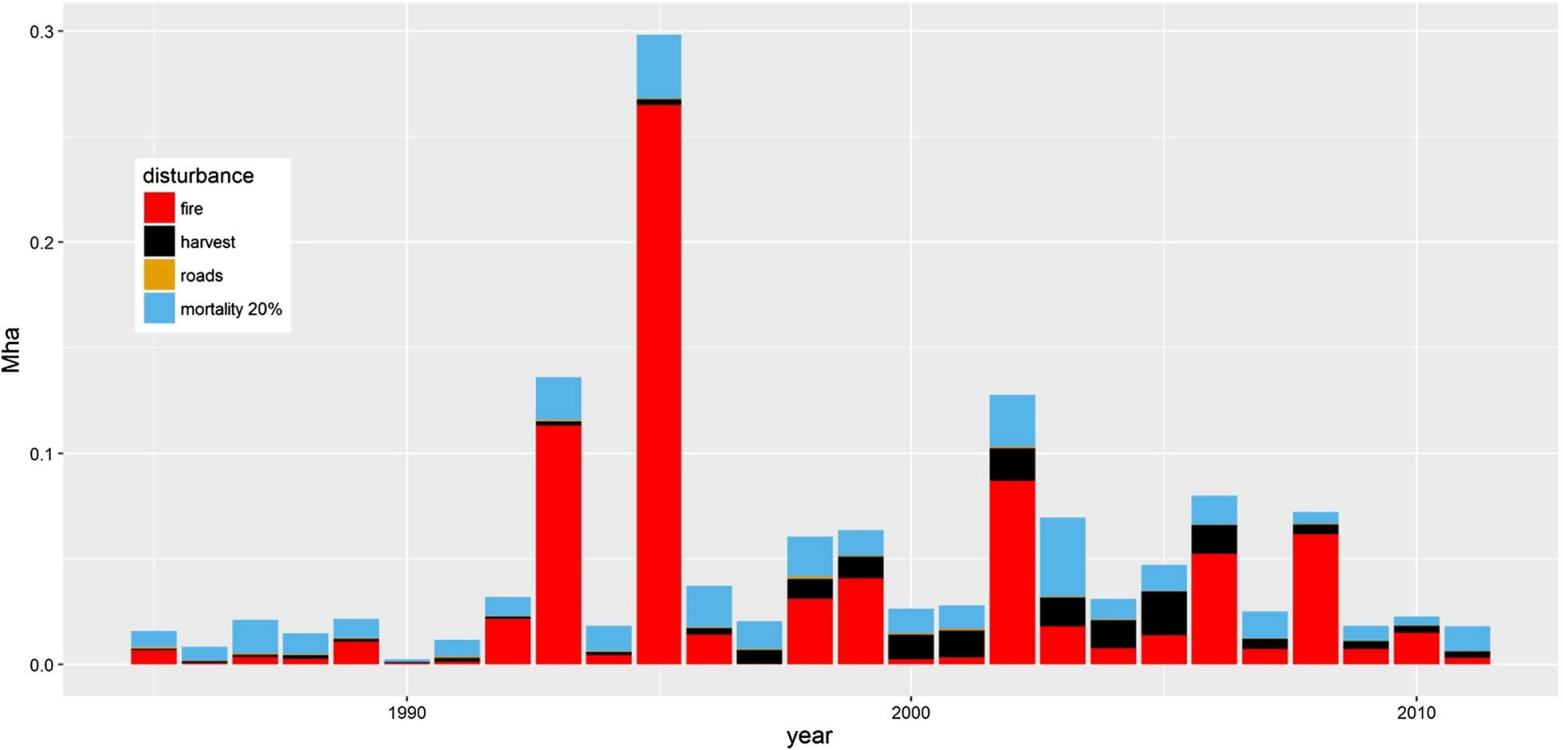
Disturbance type per year for each year of our simulation as estimated by Hermosilla et al. [14] using Landsat time series imagery

The Hermosilla et al. [14] composite2change (C2C) algorithm identifies disturbances at a 30 m spatial resolution, for the period 1984–2012 for the forested area of Saskatchewan. This disturbance type attribution differentiated five disturbance types: fire, harvesting, road, non-stand-replacing changes and unspecified. For our simulations, these disturbance types were assigned a CBM-CFS3 disturbance matrix that partitions the C transfers associated with each disturbance type among CBM-CFS3’s 21 C pools, the atmosphere, and the forest product sector. For example, a “fire” event is a stand-replacing disturbance that causes specific C-pool transfers defined by a set of parameters that vary by location (province, forest management area, and ecozone). Fires in our simulations were assigned a wildfire disturbance matrix that was pre-defined in CBM-CFS3 and is specific for the ecozones of Saskatchewan. This was the same matrix used for representing fires in the current reporting system for this area. For harvesting events, the disturbance type assigned was clearcut with salvage, specific to the practices in Saskatchewan where 85% of the merchantable trees and 50% of the snags are transferred out of the forests to the forest products sector, and logging residues are left on site to decompose over time. Areas identified as road construction were considered a deforestation event during which salvage, uprooting, and burning occur. In the absence of any other knowledge of the disturbances that were non-stand replacing, we assigned C-redistribution schema where 20% of the forest died, a relatively common disturbance event in this area which is often associated with an insect-caused defoliation event. A fifth disturbance type of unknown origin, while identified as a change event in Hermosilla et al. [14], was left unclassified due to insufficient class membership information, and also assigned a generic 20% mortality. Like in other CBM-CFS3 simulations, the effects of background endemic insect infestations are captured in forest inventory and growth increment data. The number of hectares disturbed in our simulations, by disturbance type and year, is displayed in Fig. 3.

## Results

**Fig. 4:**
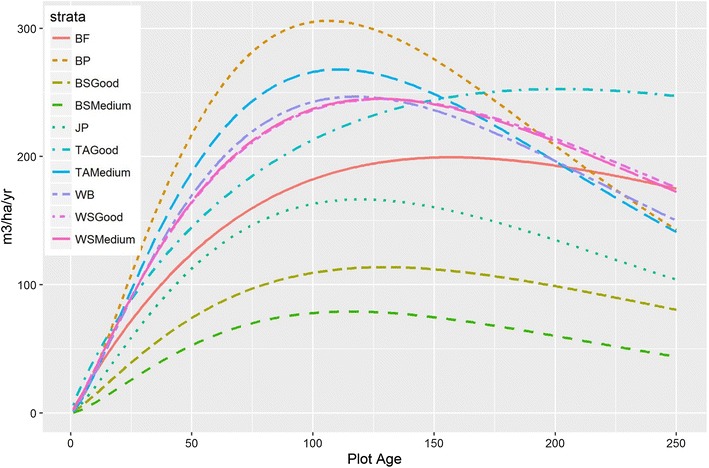
Growth* curves* used for CBM-CFS3 simulations for our test area in the managed forest of Saskatchewan, Canada

The growth curves resulting from fitting the permanent sample plot data to Hoerl’s equation (Eqs. 1a, b) are presented in Fig. 4. Stratifying the fitting data into leading species and, for some species, productivity levels obtained from CASFRI improved the model fit when applied to the intercept and first slope only. This led to nine growth curves, one per species for five species (BF, BP, JP, TA, WB), and two for each of white and black spruce representing a medium and a good productivity level for each spruce species (BSMedium, BSGood, WSMedium, WSGood). The model showed no trend in model residual and random effects were indeed normally distributed (results not shown) supporting the assumption of independent error terms, and normally distributed random effects. The curves presented in Fig. 4 were used to predict forest growth in our CBM-CFS3 simulations for our study area in Saskatchewan.

Our simulations estimate C stocks in our study area to have increased by 17.98 Tg C between 1984 and 2012, starting with an average C density of 204.85 Mg C ha^−1^ in 1984, and ending with 207.86 Mg C ha^−1^ in 2012. Over the course of the simulations, C increased in total biomass, soil C remained almost the same with a small gain, while the deadwood and snag C pools experienced losses, and the litter C pool increased.

**Fig. 5:**
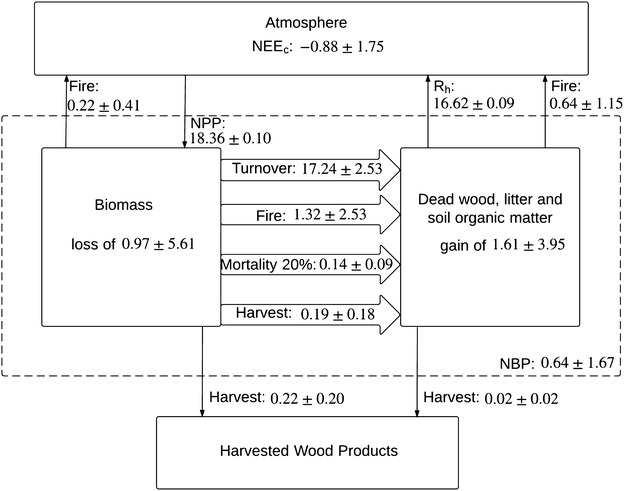
Annual means and standard deviations for C fluxes in Tg C year^−1^ for our study area in the boreal forests of Saskatchewan Canada as simulated by CBM-CFS3 on a 30-m pixel resolution between 1984 and 2012. Net ecosystem exchange (NEE) is the net amount of C removed from the atmosphere, net primary productivity (NPP), is the C absorbed by plants, Rh is the heterotrophic respiration of the system, and net biome productivity (NBP) is the total C budget of the system including disturbances. Harvest estimates include all harvest, even that which is associated with road building

Net primary productivity (NPP) represents the amount of CO_2_ removed from the atmosphere by the forest after the (autotrophic) respiration necessary to maintain itself. Some of that NPP is released as ecosystem respiration (decomposition, Rh), resulting in net ecosystem productivity (NEP), the amount of C absorbed per year by an entity such as a forest stand. Net biome productivity (NBP) is the landscape-level C balance: the sum of all stand-level NEP minus C losses due to disturbances (some losses go to the atmosphere and some to harvested wood products [55]). In Fig. 5 we present the resulting landscape-level C balance based on our simulations, with allocations and flows shown.

**Fig. 6:**
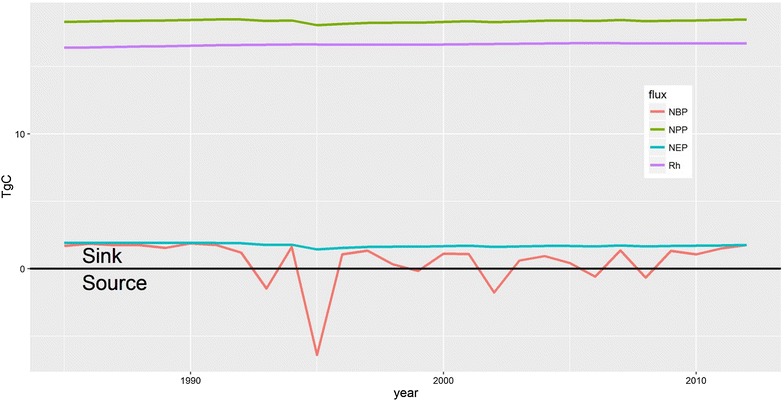
Net primary productivity (NPP—in *green*), ecosystem respiration (Rh—in *purple*), net ecosystem productivity (NEP—in *teal*), and net biome productivity (NBP—in *red*) for our study area as simulated by CBM-CFS3 between 1984 and 2012 (Tg C year^−1^) at 30 m spatial resolution. NBP includes harvested C that leaves the system

Figure 6 displays the C fluxes through time from an ecosystem perspective. All the C fluxes presented in Figs. 5 and 6 are for the forest in the entire study area over the simulation time horizon. Years where NBP (red line) dips below the 0 line in Fig. 6 are years where the landscape was a source to the atmosphere while the years above the line are years of C sink from the atmosphere. Figures 5 and 6 show that the study area is overall a C sink, with some years where the region is a source to the atmosphere, and that the largest fluxes associated with vegetation productivity (NPP) and heterotrophic respiration (Rh).

**Fig. 7:**
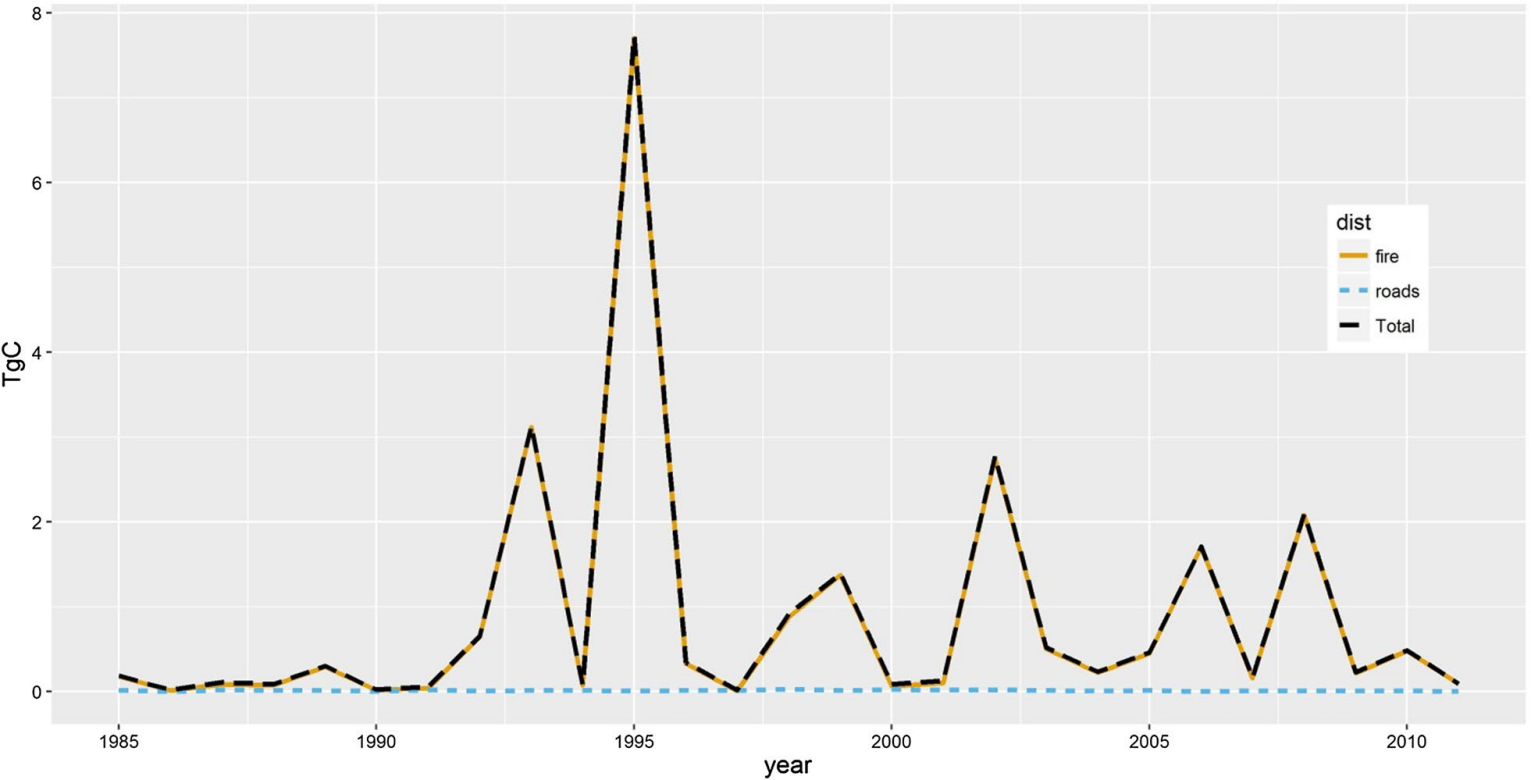
Total direct emissions in Tg C year^−1^ (*black line*) to the atmosphere from stand-replacing disturbances (fire in *yellow*, roads in *blue*) between 1984 and 2012 for our study area. The *coloured lines* show the contribution of each disturbance type to the total emissions

In CBM-CFS3, C pools describe the C content of live forest components (above and below ground biomass), the C content of the dead components (also above and belowground, including litter, deadwood and soil), an atmospheric C pool for transfers to and from the atmosphere, as well as a pool for C leaving the system to the forest product sector. The C emissions due to decomposition, shown in Fig. 6 (Rh, in purple), are the sum of the C that moves from the various dead C pool into the atmosphere. Disturbances dictate a transfer of C from specified pools to other specified pools, for example, the 20% mortality disturbance we used in our simulations transfers a portion of the C in live biomass to the dead organic matter pools where it will decay over time, hence, slowly release C to the atmosphere (Rh). The total direct emissions to the atmosphere resulting from fire and road building are presented in Fig. 7. Lateral transfers of biomass to dead organic matter or of biomass to the forest product sector are depicted in Fig. 5.

**Fig. 8:**
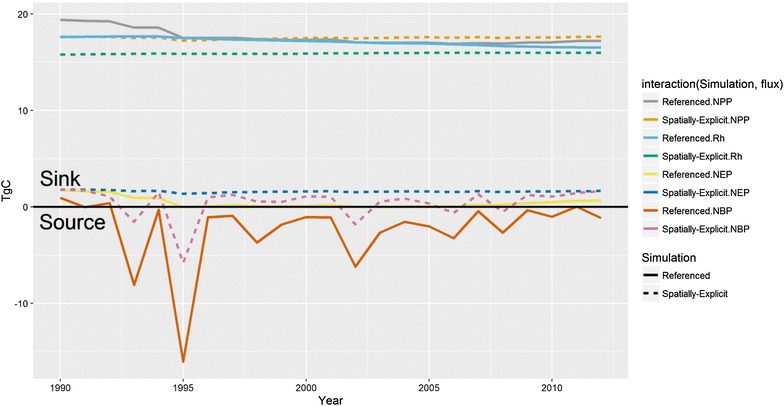
C fluxes in Tg C year^−1^ over the whole simulated landscape as estimated for the spatially-referenced simulation (*solid lines*) under the UNFCCC and from our spatially-explicit simulation (*dotted lines*) for forest areas in Saskatchewan, Canada. Negative values of NBP (*solid orange line* for the spatially-referenced simulations, and *dotted pink line* for the spatially-explicit simulations) depict a source of C out of the system (emissions to the atmosphere + C harvested)

The same general trends have been detected in the current 1990–2013 GHG reporting system for this region [41], where years with a large area burned (e.g. 1995) produced a concomitant large pulse of GHG emissions into the atmosphere. Direct comparison between the Saskatchewan portion of the UNFCCC reported values (hereafter, spatially-referenced simulations) and our analyses (hereafter, spatially-explicit simulations) was difficult, in part because of a discrepancy in what is counted as forested area. In the reported values from the spatially-referenced simulations, the number of hectares that are identified as forest and remain forest is defined by land classification and may include non-treed areas recently disturbed but classified as forest land. In contrast, the photo-interpreted characteristics that are the basis for defining forest area from the spatial inventory are defined by what the photointerpreter identifies as forest, which does not necessarily include the areas that are regenerating from either fire or harvest. In the area of 100% overlap between the spatially-referenced and spatially-explicit simulations, the spatially-explicit simulations account for 86% of the total area included in the spatially-referenced simulation. In this overlap area, there is a difference in stand-level C density between the spatially-referenced (324.1 Mg C ha^−1^) and spatially-explicit (358.1 Mg C ha^−1^) simulations. Although the C stocks are important for estimating the potential GHG release, GHG fluxes are more relevant for the short reporting periods used herein. For the same area and overlapping simulation years (1990–2012), the spatially-referenced simulations, which were part of UNFCCC reporting, estimated a total cumulative C source of −66 Tg C, while our spatially-explicit simulations estimated a total cumulative C sink of +7 Tg C. Figure 8 compares the annual fluxes estimated for both simulations.

## Discussion

**Fig. 9:**
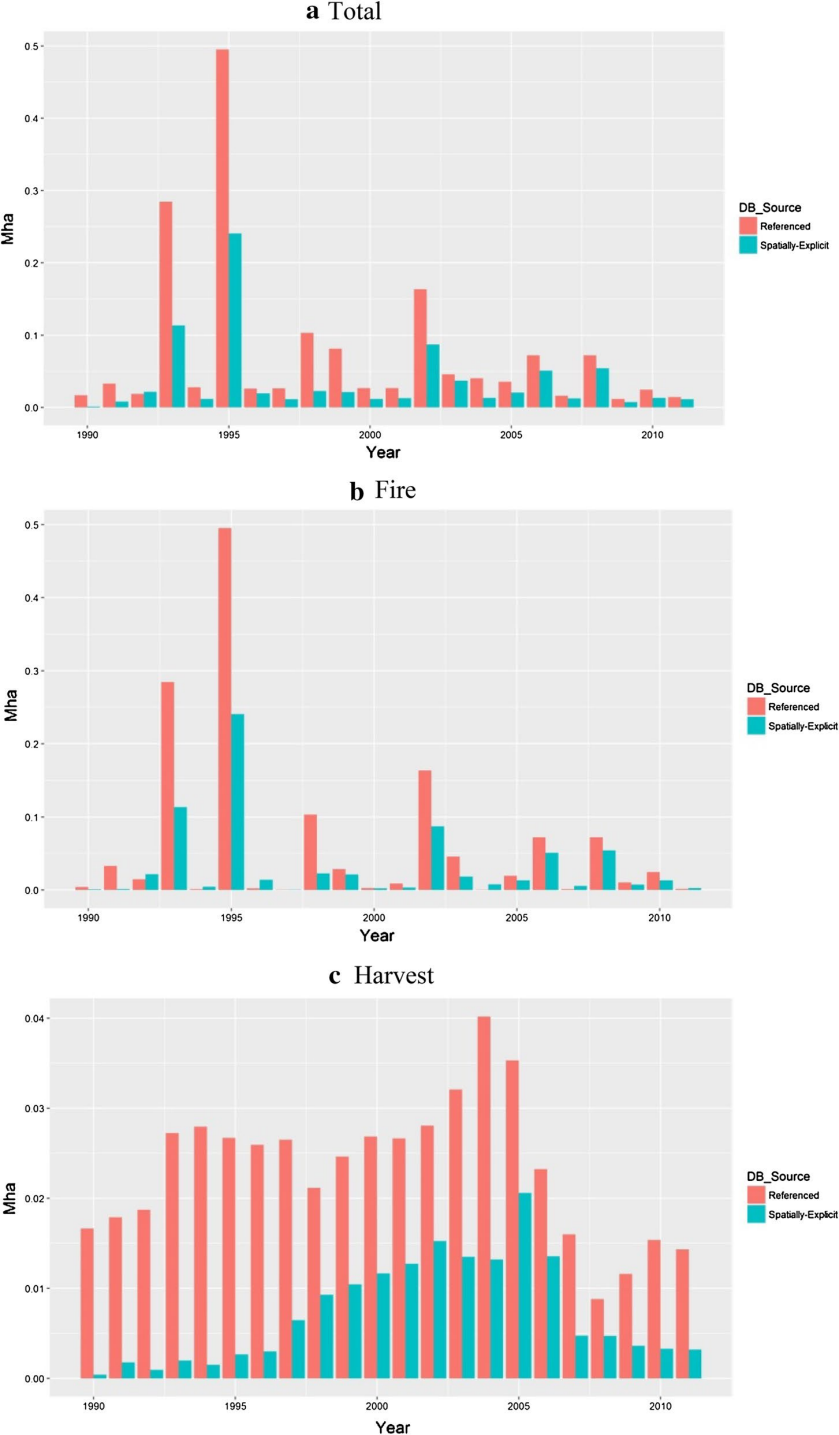
Area disturbed per year in CBM-CFS3 simulations: in *pink*, estimates from the spatially-referenced simulations used for reporting purposes, and in *blue*, from our spatially-explicit simulations for **a** all disturbances combined, **b** for areas disturbed by fire only, and **c** for areas disturbed by timber harvesting

Accurate estimates of GHG exchanges in the boreal forest are required under international reporting commitments and for climate change mitigation and policy development. GHG emissions and removals estimation for forests is more complicated and varied than estimates from many industrial sectors where emissions can often be directly measured. In boreal forests, where disturbances often drive emissions variability, temporally and spatially-explicit disturbance identification at a resolution that captures forest management practices at the stand level offers a new opportunity to improve GHG balance estimates. We are aware of only one other published effort at incorporating this type of data into GHG reporting procedures, which is applied to the Yucatán peninsula of Mexico [19, 33], a system where emissions and removals of C are dominated by smaller scale disturbances, posing a different problem than that in the boreal forests. Figure 7 confirms that fire is the main contributor to direct C emissions for our study area. Comparing total emissions (black line Fig. 7) to NBP (red line Fig. 9), we can see that the years with high occurrence of fires are the years that the system as a whole was a C source to the atmosphere despite NBP including the harvested C leaving the system but not emitted to the atmosphere. Notwithstanding these years of high fire occurrence and a shift in age-class distribution, which switches from more younger forest stands with high C uptake forests in 1984, to older forests with slower C uptake forests in 2012 (see Fig. 2), the forest of our test region was a cumulative C sink of 17.98 Tg C over the simulation period.

When compared to the spatially-referenced simulations used for UNFCCC reporting, the landscape-level C balance (NBP in Fig. 8) shows the same general patterns (synchronized peaks and valleys) in the reported values as our spatially-explicit results. However, the spatially-referenced simulations depict the area as a C source to the atmosphere (i.e., it is mostly below the 0 line), while the spatially-explicit simulations show the area as a sink. Thus, despite the forest area in our spatially-explicit simulations having fewer forested hectares (14% less) than the spatially-referenced simulations, the large fluxes (Rh and NPP) are in the same range, with the difference that the overall productivity (NEP) in our spatially-explicit simulations is greater than respiration. More productive forests tend to store more C in boreal systems and recently disturbed forests areas tend to be C sources for 10–20 years post disturbances [6], which both contribute to the differences in absolute C leaving the system. The causes of our spatial simulations having higher NPP over Rh could result from differences in disturbance and growth rates, as well as age-structure, all of which differ between the simulations. We explore these differences in the following text.

The area disturbed by year is compared in Fig. 9 for (a) the total area disturbed, and the two most important disturbances, fire (b) and harvest (c). For comparison purposes the larger number of disturbance types in the spatially-referenced simulations were lumped into four categories according to the four disturbance types used in our spatially-explicit simulations.

The cumulative difference in hectares disturbed between the spatially-referenced and our spatially-explicit simulations for the period 1990–2011 is 924,924 ha. Of that, 56% is due to differences in area burned (fire), and 29% is due to a difference in area harvested. In both cases, the spatially-referenced simulations disturbed more hectares overall than our spatially-explicit simulations, contributing to a large proportion of the differences in the C balance. The number of hectares disturbed in the spatially-referenced simulations is based on provincial statistics and national compilations for both fire and harvest (see [34]). Fires are the predominant disturbance on the landscape, and years of large fires are detected in both simulations. For fires, these burned-area compilations use both coarse-resolution (e.g., SPOT-VGT) and medium-resolution remotely-sensed data sources which include Landsat information, as well as other sources, including aerial GPS surveys and manual delineation from air photos. In some cases, unburned islands or water bodies found within fire perimeters are not removed, but rather included in area burned totals. This would contribute to differences in the total area burned. The Landsat change product used in our spatially-explicit simulations detected, in most years, less area burned than the data used in the spatially-referenced simulations, primarily as a result of differences in the data, spatial resolution, and methods used for fire detection and mapping. With increasing spatial resolution, greater detail is captured with less generalization of disturbance edges [56] and improved capture of within-fire boundary variability, such as unburned islands [38]. For harvest, much less area was estimated as harvested by the Landsat change product relative to the harvest data used in the spatially-referenced simulation. For the latter, harvest area is allocated from aspatial provincial summaries. All of these differences contribute to the differences we see in our comparison: less area disturbed contributes to lower emissions and the maintenance of higher C stocks on the landscapes, more recently-disturbed stands act as C sources for 10–20 years post disturbance.

Further, in the same overlap area, the Landsat change product identified disturbances occurring between 1990 and 2011 for areas that were not modeled due to a lack of corresponding forest inventory data for those areas. Notwithstanding that the spatial forest inventory used in our spatially-explicit simulations could likely be further refined, since photo-interpreted inventories have error that is estimated to be in the range of 20–30% for individual attributes [50], it would be difficult to determine if those disturbed, but not modelled hectares contribute to the 14% difference in forested area (i.e., hectares considered forested under the spatially-referenced simulations that had no corresponding data in our spatial inventory), or if they are part of the mix between wetland/peatland and forests that may have been disturbed but is not classified as forests. These areas will therefore contribute to a reduced C sink in the spatially-referenced simulations. In the non-modelled category of disturbed ha (0.07%), the majority falls in the non-stand-replacing condition (42%) and another portion in the unclassified disturbance (20%); in this non-modelled category there is very little harvest or roads (1.25%), with some fire (29%). This may indicate that these areas represent less productive forests. Also, >60% of hectares in this category would have been modelled as a 20% mortality disturbance, with low impact on the short-term C-balance.

We are aware that assigning a 20% mortality C-redistribution scenario to the low disturbance condition and to the unclassified disturbance is a simplification of the forest dynamics in boreal forests. This simplification results in 26% of the disturbed area being assigned a 20% mortality scenario, more than any individual disturbance, apart from fire or harvest, would have in the forests of Saskatchewan. This is a consequence of the relatively coarse attribution of forest change to disturbance types. The origin of much of the non-stand replacing disturbance remains to be determined. The proximity of much of this class to surficial water may suggest that it represents the ecotone between peatlands/wetlands and forests that is pervasive in much of the boreal forests [57]. Considering the low impact on the C-balance of this category, the relatively small area impacted by this (0.06% of the modelled forested hectares), and that further refining this category is not presently possible, we assess the consequences on our estimated C-balance of this generalization to be minimal. It may also be the case that these types of changes are difficult to specify at a 30 m resolution [58]. Deforestation events in general (other than roads) are rare in this part of Saskatchewan and would have little influence on the differences in C dynamics that we are exploring. Further, the Landsat disturbance product uses an agricultural mask in 2011, and would therefore not track swaps between forested lands and agricultural lands before that date, something the spatially-referenced simulations are designed to track. The spatially-referenced simulations simulated 44,849 ha of deforestation in the comparison area between 1990 and 2012.

Disturbance types used in UNFCCC reporting spatially-referenced simulations were more diverse than those depicted in our spatially-explicit simulations, as the Landsat change product only identifies four types of disturbances. The initial change/no-change attribution of stand replacing disturbance is however, of a high accuracy (92.2%). It is also worth noting that of these four types that could be reliably labeled, two types represented >72% of the area disturbed, had a reported area level accuracy of 98%, and are the dominant drivers of C balance in boreal forests. While refinement of the Landsat-based attribution could improve the C-balance estimate further, the attribution task is not a simple one [14, 33, 59]. Some understanding of the disturbance types in a given region will dictate the importance of capturing particular classes. The high accuracy of stand replacing change detection, as well as the harvest and wildfire categories, allows for additional focused investigation to allocate types to remaining changes, such as for the attribution of deforestation and the related land-use and land-cover transitions involved. Insect infestations are harder to detect [44] yet are known cover large areas of the Canadian forests and impact the C balance ([60]—mountain pine beetle, [61]—spruce bud worm), while in other jurisdictions, human activities dominate the C balance [19, 33]. Hence, improvements in the attribution of the change detected via remote sensing are desirable, and research in this domain is ongoing [62, 63].

Another contribution to the differences in C-balance between the spatially-referenced and spatially-explicit simulations (for the overlap area) is growth rates. Growth estimates that drive our spatial CBM-CFS3 simulations were directly developed from permanent sample field plots which are the best available data on growth [9], and using mixed-effect models that take into account the data structure, hence, proven statistical methods [52]. The growth information used for the GHG reporting spatially-referenced simulations in this region relied on 15-year old compilations of fewer plots, not specifically designed to monitor growth, with poor fitting methods (see, [34]), and hence, were less specific to the target area. A cursory comparison of the two sets of growth estimates showed that the growth rates depicted by the field plots (Fig. 4) used in our spatial simulations are higher than those used for the spatially-referenced simulations. More productive forests, those sites that are able to carry more C per ha, tend to remove more CO_2_ from the atmosphere, further contributing to differences in productivity. Further, in the CBM-CFS3 simulations, growth curves are used to initialise C pools, hence, from the starting point, our spatial simulations had more C in the system, or at least more C concentrated on the hectares that were modelled, than the reported GHG balance for the area. One could argue that selecting the most productive sites for estimating growth across the landscape, as permanent sample field plots do, may not reflect the reality of forest growth conditions across the landscape, and this is an area of active research that we are exploring.

## Conclusions

The goals of our study were to advance C-balance modelling towards spatially-explicit systems and to evaluate the impact of these new approaches on the estimates of the C balance of a boreal forest landscape. Our simulations revealed that a 30 m resolution brings into focus the heterogeneity of the landscape, and that although the general fluxes have similar patterns (valleys and peaks) to those reported, C density and sink estimates are higher than those reported. Mascorro, Coops [19] analysed the impacts of four remote sensing products on GHG balance estimates and also found that using different remotely-sensed data for determining disturbances yielded different estimates of C fluxes over their study area in Mexico.

Using spatially-explicit disturbance information in combination with a spatial forest inventory will improve the characterization of pre-disturbance forest conditions (age, volume, fuel loading) and should therefore help to reduce uncertainties in GHG emissions and removals estimates that are inherent in the current spatially-referenced approaches. Another benefit of spatially-explicit modelling is the potential to better inform post-disturbance productivity information based on pre-existing stand-specific conditions. There are still, however, many aspects of C balance estimation that can be improved.

The sparsity of field data is one of the main impediments to many possible improvements to C balance estimates. In the Canadian case, spatial forest inventories are developed by provincial/territorial jurisdictional resource management agencies, with the extents, attribution content, and time periods represented intended to meet strategic information needs [64]. As a result, the data are of variable vintage, spatial coverage, scales, and information content. The current version of CASFRI which covers most of the managed boreal forests of Canada and is the input spatial inventory for our study region, comprises 36 source datasets and contains 25,319,505 polygons covering a total area of 3,635,970 km^2^. The minimum mapping unit ranges from 1 to 8 ha, comparable with the resolution of many remotely-sensed land-cover products. In most inventories, smaller identifiable features such as small patches of a distinct tree species are incorporated into the description of the larger unit in which they lie. Refinement and update of spatial inventory inputs with a more direct link to current field inventories will be essential in the production of reliable GHG balance estimates for forests.

Field and remote sensing data could also inform the post-disturbance productivity or stand dynamics. CBM-CFS3 can accommodate such information but data were not available at the appropriate scale to inform post-disturbance transition in our simulations, nor were they available for the current GHG reporting simulations. Refining the post-disturbance succession trajectories and productivity would greatly improve the accuracy of the C balance, and may permit the incorporation of the climate change effects on species dynamics. Remote sensing is well poised to contribute information to these efforts [65–68], although field data would still be necessary to support remote sensing observations.

Present reporting requirements use the IPCC “managed land proxy” and limit reporting to anthropogenic emissions, which are defined as those occurring within the managed portion of the forested systems. However, for scientific reasons and to understand the contribution of Canada’s forests to the global C cycle, Canada also plans to estimate the stocks and fluxes of all its forests. Efforts are presently underway in Canada and elsewhere to expand GHG balance estimates to include managed and unmanaged forests.

This study is a step towards comprehensive spatially-explicit forest C estimation and reporting, with insights relevant for practices implemented in Canada and elsewhere. The full implementation of a second generation National Forest Carbon Monitoring, Accounting and Reporting System will require further developments of the modelling environment to enable the processing of the entire spatially-explicit time series of disturbances that is now available from Landsat satellites and, in the future, could be augmented by measurements from other operational satellites [69]. However, building models and tools that enable explicit links between earth observation products and estimates of GHG emissions is a much needed effort that will enhance the capabilities of future monitoring, reporting and verification (MRV) systems for the land use, land-use change and forestry sector, that can also be used in support of efforts to reduce emissions from deforestation and degradation (REDD) in developing countries.

## Abbreviations

C: carbon
GHG: greenhouse gas
CBM-CFS3: Carbon Budget Model of the Canadian Forest Sector
CO_2_: carbon dioxide
IPCC: Intergovernmental Panel on Climate Change
GEO: Group on Earth Observations
GEOSS: Global Earth Observation System of Systems
GFOI: Global Forest Observations Initiative
UNFCCC: UN Framework Convention on Climate Change
FAO: Food and Agriculture Organization
FRA: forest resources assessment
NFCMARS: Canada’s National Forest Carbon Monitoring, Accounting and Reporting System
BF: balsam fir (*Abies balsamea)*
BP: balsam poplar (*Populus balsamifera*)
BS: black spruce (*Picea mariana*)
JP: jack pine (*Pinus banksiana*)
TA: trembling aspen (*Populus tremuloides*)
WB: white birch (*Betula papyrifera*)
WS: white spruce (*Picea glauca*)
CASFRI: Canada’s Forest Resource Inventories
C2C: composite2change
BSMedium: black spruce medium productivity
BSGood: black spruce good productivity
WSMedium: white spruce medium productivity
WSGood: white spruce good productivity
NPP: net primary productivity
Rh: heterotrophic respiration (decomposition)
NEP: net ecosystem productivity
NBP: net biome productivity
NEE: net ecosystem exchange
MRV: monitoring, reporting and verification
REDD: reduce emissions from deforestation and degradation
NTEMS: National Terrestrial Ecosystem Monitoring System
CSA: Canadian Space Agency
GRIP: Government Related Initiatives Program
CFS: Canadian Forest Service
